# Temporal Dynamics of the Integration of Intention and Outcome in Harmful and Helpful Moral Judgment

**DOI:** 10.3389/fpsyg.2015.02022

**Published:** 2016-01-11

**Authors:** Tian Gan, Xiaping Lu, Wanqing Li, Danyang Gui, Honghong Tang, Xiaoqin Mai, Chao Liu, Yue-Jia Luo

**Affiliations:** ^1^State Key Laboratory of Cognitive Neuroscience and Learning & IDG/McGovern Institute for Brain Research, Beijing Normal UniversityBeijing, China; ^2^Department of Psychology, Zhejiang Sci-Tech UniversityHangzhou, China; ^3^Center for Collaboration and Innovation in Brain and Learning Sciences, Beijing Normal UniversityBeijing, China; ^4^Department of Psychology, Renmin University of ChinaBeijing, China; ^5^Institute of Affective and Social Neuroscience, Shenzhen UniversityShenzhen, China; ^6^Collaborative Innovation Center of Sichuan for Elder Care and Health, Chengdu Medical CollegeChengdu, China

**Keywords:** morality, event-related potential (ERP), integration of intention and outcome, harmful moral judgment, helpful moral judgment, temporo-parietal junction

## Abstract

The ability to integrate the moral intention information with the outcome of an action plays a crucial role in mature moral judgment. Functional magnetic resonance imaging (fMRI) studies implicated that both prefrontal and temporo-parietal cortices are involved in moral intention and outcome processing. Here, we used the event-related potentials (ERPs) technique to investigate the temporal dynamics of the processing of the integration between intention and outcome information in harmful and helpful moral judgment. In two experiments, participants were asked to make moral judgments for agents who produced either negative/neutral outcomes with harmful/neutral intentions (harmful judgment) or positive/neutral outcomes with helpful/neutral intentions (helpful judgment). Significant ERP differences between attempted and successful actions over prefrontal and bilateral temporo-parietal regions were found in both harmful and helpful moral judgment, which suggest a possible time course of the integration processing in the brain, starting from the right temporo-parietal area (N180) to the left temporo-parietal area (N250), then the prefrontal area (FSW) and the right temporo-parietal area (TP450 and TPSW) again. These results highlighted the fast moral intuition reaction and the late integration processing over the right temporo-parietal area.

## Introduction

The ability to integrate mental states such as intentions with outcome information plays an important role in moral judgment. People judge attempted harms (e.g., a person who intends to kill someone but failed) as less permissible and more morally blameworthy than accidental harms (e.g., a person accidentally kills someone) (Young et al., 2007; Young and Saxe, 2008). Recently, the integration of intention and outcome in moral judgment has been systematically investigated (Young et al., 2007, 2011; Young and Saxe, 2008, 2009). In these experiments, participants read scenarios in a 2 × 2 design: agents produced either a negative or neutral outcome while intending either the negative outcome (“negative” intention) or the neutral outcome (“neutral” intention). Thus, the combination of intention and outcome yielded four conditions: successful harm, attempted harm, accidental harm and no harm. Behavioral results of healthy adults demonstrated a significant interaction between intention and outcome (Young et al., 2007, 2011, 2012). These findings reveal that mature moral judgments depend crucially on the integration processing between an agent’s intention and actual results of action.

Functional magnetic resonance imaging (fMRI) studies investigating the processing of moral intention and outcome have revealed a network of brain regions, including the medial prefrontal cortex, precuneus and temporo-parietal junction (TPJ) (Young et al., 2010; Young and Dungan, 2012). However, the temporal dynamics of this network is still unknown. Particularly, some studies found right TPJ activation during theory of mind (TOM), mentalizing and integration processing (Saxe and Kanwisher, 2003; Young et al., 2007, 2010), others reported right TPJ activation associated with moral intuition (Harenski et al., 2010). These fMRI findings implied that right TPJ activation may be involved in both early moral intuition and late moral reasoning processing during the integration of intention and outcome. However, the time course of this integration processing at right TPJ are poorly understood.

Against this background, the first aim of the present study was to investigate the electrophysiological mechanisms of the integration between moral intention and outcome using event-related potentials (ERPs). The measurement of ERPs provides an excellent method to investigate the temporal features of information processing in moral cognition due to the high temporal resolution available from the ERP signal. Previous ERP studies of moral judgment focused on the interaction between cognition and emotion in moral processing (Chen et al., 2009; Sarlo et al., 2012, 2014; Wang et al., 2014; Gui et al., 2015). For instance, in our previous study, we found the larger N1 for moral pictures than non-moral pictures, which may reflect the early moral intuition processing without the emotional impact. For mental states attribution, Proverbio and Riva, 2009; Proverbio et al., 2010) reported an enhanced posterior negative component that peaked around 250 ms (N250), which reflected the early recognition of comprehensible behaviors and the early processing of action’s purpose. Another study further suggested that the N250 may reflect the early cognitive processing to understand private intention (Wang et al., 2012). During the late time windows (from 300 to 800 ms), slow wave effects over frontal and parietal areas were reported to reflect specific cognitive process of monitoring others’ beliefs (Liu et al., 2009; McCleery et al., 2011; Geangu et al., 2013). Specially, McCleery et al. (2011) found a positive component over bilateral temporo-parietal areas peaking between 400 and 500 ms (TP450) and suggested that this component could reflect the calculating and representing processes in the visual perspective taking. However, most of these studies focused only on the mental states reasoning during the classical TOM tasks, few have investigated the intention information processing in moral judgment. So far, only three ERP studies investigated the neural correlates of moral intention and valence processing. The results reported significant ERP effects indicating early automatic and late controlled processes (Van Berkum et al., 2009; Decety and Cacioppo, 2012; Yoder and Decety, 2014). Decety and Cacioppo (2012) found that people could distinguish between intentional and accidental harm in as fast as 62 ms post-stimulus. Van Berkum et al. (2009) reported a rapid ERP response to the first word that indicated a clash with the reader’s value system within 200 ms. And significant differences on N1 amplitude between morally good and bad actions were reported by Yoder and Decety (2014). However, tasks used in these studies were only related to intention decoding or moral valence, and the processing of integrating intention with outcome information has not been considered yet. Exploring the time course of integration between intention and outcome may reveal different integration processing stages in moral judgment.

The second aim of the present study was to explore the cognitive and neural mechanisms of helpful moral judgment. Helpful behaviors are critically important for human social development (Graham, 2014; Hofmann et al., 2014). However, most moral neuroscience studies have concentrated on immoral and negative behaviors such as killing, murder and harm (Greene and Haidt, 2002; Young et al., 2007; Greene, 2009; Graham, 2014). Only a few studies have explored the neural processing during moral judgment of helpful behaviors (Loke et al., 2011; Young et al., 2011; Paulus et al., 2012; Yoder and Decety, 2014). The neural correlates of helpful intention processing, especially the integration of helpful intention and outcome in moral judgment is thus left to be explored. Previous studies have found that humans respond differently to negative and positive information (Baumeister et al., 2001). For instance, when people make decisions, they typically exhibit greater sensitivity to losses than to equivalent gains, which is called loss aversion (Tom et al., 2007). Is there a similar asymmetry between the moral judgment of good and bad actions? Will the judgments of helpful behaviors be different from the judgment of harmful behaviors? In order to explore these questions, in addition to harmful moral processing, positive intention and outcome processing in helpful moral judgment were also considered in the present study.

Based on previous work, we used ERPs to investigate the temporal dynamics of the integration processing of intention and outcome information of harmful and helpful moral judgment in two experiments. We predicted that: (a) In accord with previous findings (Van Berkum et al., 2009; Decety and Cacioppo, 2012; Yoder and Decety, 2014; Gui et al., 2015), the fast moral intuition might be revealed by the early ERP effects which are sensitive to the successful harm and help. Besides, late ERP effects in accidental and attempted conditions might reflect the integration in late processing stage; (b) According to the fMRI studies which reported the significant activation of right TPJ, left TPJ and mPFC during the integration of intention and outcome (Koenigs et al., 2007; Young et al., 2007; Funk and Gazzaniga, 2009; Young and Dungan, 2012; FeldmanHall et al., 2014), we predicted that ERP effects would be elicited over frontal and temporo-parietal electrodes, especially the right TPJ area; (c) The previous fMRI studies have found similar brain activation in judging harmful vs. helpful actions (Young et al., 2011). Based on these findings, we predicted that the temporal processing stages of the integration of intention and outcome in harmful and helpful moral judgment would be similar, which would be shown by similar ERP patterns in the current two experiments. However, the interaction effects between intention and outcome of ERPs would be different in harmful and helpful moral judgment because of the possible asymmetry between positive and negative processing (Baumeister et al., 2001).

## Materials and Methods

### Participants

Fifty-five undergraduate students from Beijing Normal University participated in the study. Twenty-seven students (12 males, *M*_age_ = 22.8 years, *SD* = 2.1) participated in Experiment 1 and a different group of 28 students (13 males, *M*_age_ = 22.4 years, *SD* = 1.6) participated in Experiment 2. All participants were right-handed, with no history of neurological/psychiatric illness. All the experimental procedures used in Experiments 1 and 2 were approved by the institutional review board (IRB) of Beijing Normal University (School of Brain and Cognitive Sciences) and informed written consent was obtained from each participant in accordance with the Declaration of Helsinki.

### Stimuli and Experimental Procedures

**FIGURE 1:**
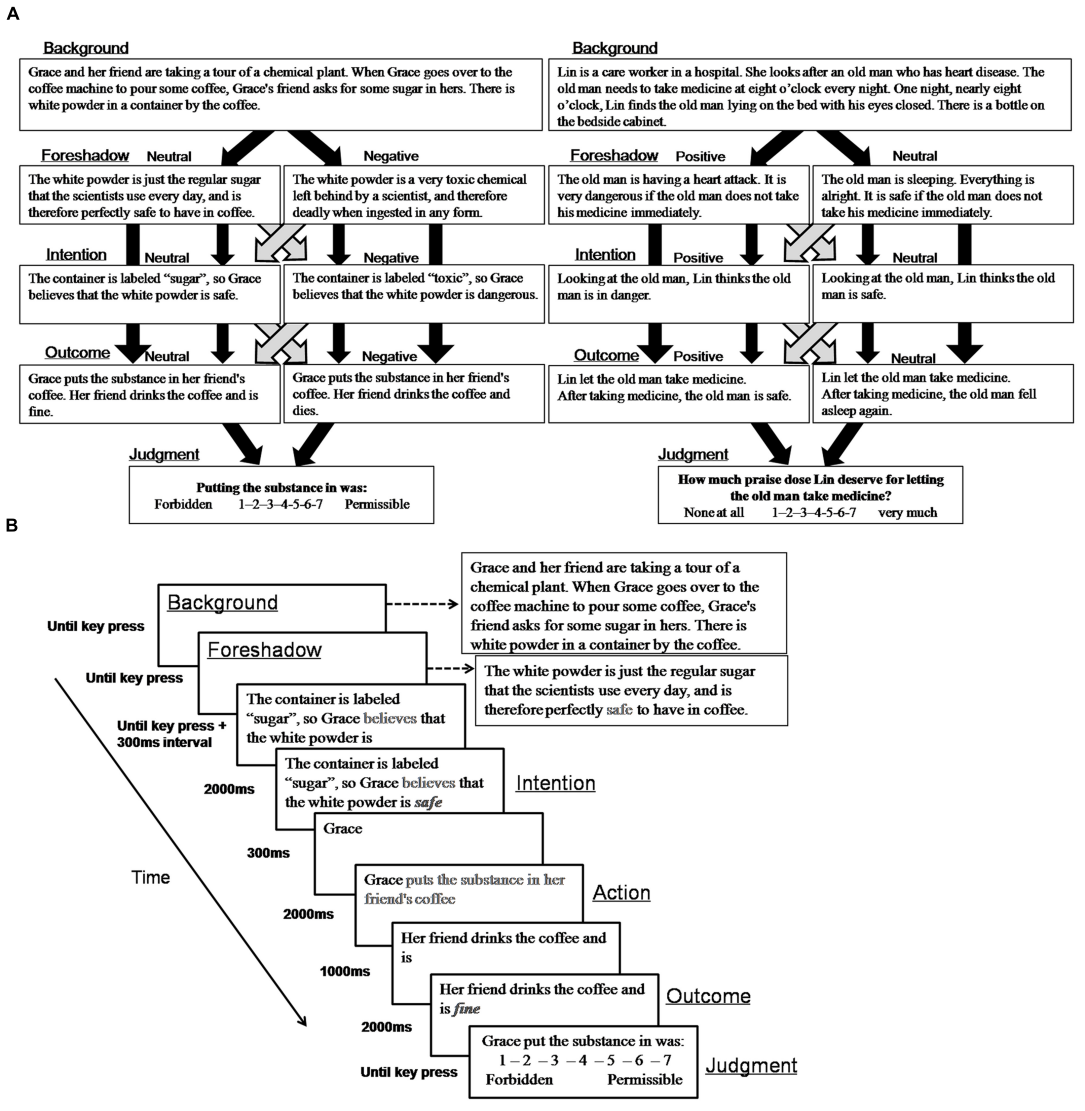
**(A)** Schematic representation of sample harm (left) and help (right) scenarios. **(B)** Schematic representation of a single moral judgment trial.

Stimuli consisted of four variations of 40 harm scenarios selected from Young’s studies (Young et al., 2010) for experiment 1 and 40 help scenarios selected from a pilot study for Experiment 2 (**Figure 1A**), for a total of 160 stories for each experiment. In the pilot study, we wrote 51 help scenarios and asked 25 students (13 males, *M*_age_ = 23, *SD* = 2.95) to evaluate how much moral praise the agent deserves for his or her action from 1 (*None at all*) to 7 (*Very much*). 40 scenarios with significant rating differences (no help, 2.61 ± 0.83; accidental help, 3.77 ± 1.16; attempted help, 5.23 ± 0.74; successful help, 6.35 ± 0.47) and high internal consistency (the Chronbach’s alpha: no help: 0.905; accidental help: 0.960; attempted help: 0.921; successful help: 0.891) were chosen as the materials used in Experiment 2. For harm scenarios, agents produced either a negative outcome or a neutral outcome with the intention that they were causing a negative outcome (“negative” intent) or a neutral outcome (“neutral” intent). For help scenarios, agents produced either a positive outcome or a neutral outcome with the intention that they were causing a positive outcome (“positive” intent) or a neutral outcome (“neutral” intent). Harmful outcomes referred to injury to others and helpful outcomes referred to saving others’ lives. Consistent with fMRI studies (Young et al., 2007), each story consisted of background, foreshadow, intention, action and outcome segments:

(1) Background: information to set the scene (identical across conditions)
(2) Foreshadow: information foreshadowing outcome (valenced or neutral)
(3) Intention: the agent’s intention about the situation (valenced or neutral)
(4) Action: the agent’s action (identical across conditions)
(5) Outcome: the actual outcome (valenced or neutral)

The experiments were conducted in a dimly lit, sound-proof room. Participants were seated on a comfortable chair with their eyes approximately 90 cm away from a 17-in computer screen. The timeline of a trial was adapted from fMRI studies (Young et al., 2007, 2011). Intention, action and outcome segments were separated for the analysis of ERPs time-locked to the key information (**Figure 1B**). In each trial, background and foreshadow information were presented cumulatively until participants pressed the space key or for a maximum of 10 s. Then, the intention segment was shown without the keywords indicating the valence of the agent’s intention. After participants pressed the space key, the keywords of intention segment (e.g., “safe”) was presented for 2 s after a 300 ms delay. The action and outcome segments were then presented cumulatively, and the keywords of the action and outcome segments were presented in a manner similar to the intention segment keywords. At the end of the trial, participants were required to judge the moral permissibility of the agent’s action from 1 (*Forbidden*) to 7 (*Permissible*) in Experiment 1, and to judge how much moral praise the agent deserves for the action from 1 (*None at all*) to 7 (*Very much*) in Experiment 2. In both experiments, following eight practice trials, participants completed 160 test trials and could take a short break after every 20 trials. The sequence of stories was pseudo-randomly ordered and no scenario was repeated in five consecutive stories.

### ERP Recordings

The electroencephalogram (EEG) was recorded from 64 scalp sites using tin electrodes mounted in an elastic cap (NeuroScan Inc.). Reference was placed at vertex by default. Horizontal electrooculogram (EOG) was recorded from electrodes placed at the outer canthi of both eyes. Vertical EOG was recorded from electrodes placed above and below the left eye. All interelectrode impedance was maintained under 5KΩ. EEG and EOG signals were amplified with a 0.05–100-Hz bandpass filter and continuously sampled at 500 Hz/channel.

During off-line analysis, EEG was re-referenced to the average reference. Ocular artifacts were removed from the EEG signal using a regression procedure implemented in the Neuroscan software (Semlitsch et al., 1986). The EEG was averaged in 1400-ms epochs (200-ms baseline) time-locked to the presentation of the keywords in the intention segment. These averages were digitally filtered with a 30-Hz low-pass filter and were baseline corrected by subtracting from each sample the average activity of that channel during the baseline period. Any trials in which EEG voltages exceeded a threshold of ±80 μV during the recording epoch were excluded from the analysis.

## Results

### Behavioral Results

Permissibility and praise judgments of harm and help scenarios, respectively, as well as reaction times were analyzed using separate 2 (intention: valence, neutral) × 2 (outcome: valence, neutral) repeated measures ANOVA:

#### Moral Judgments of Harm Scenarios

**FIGURE 2:**
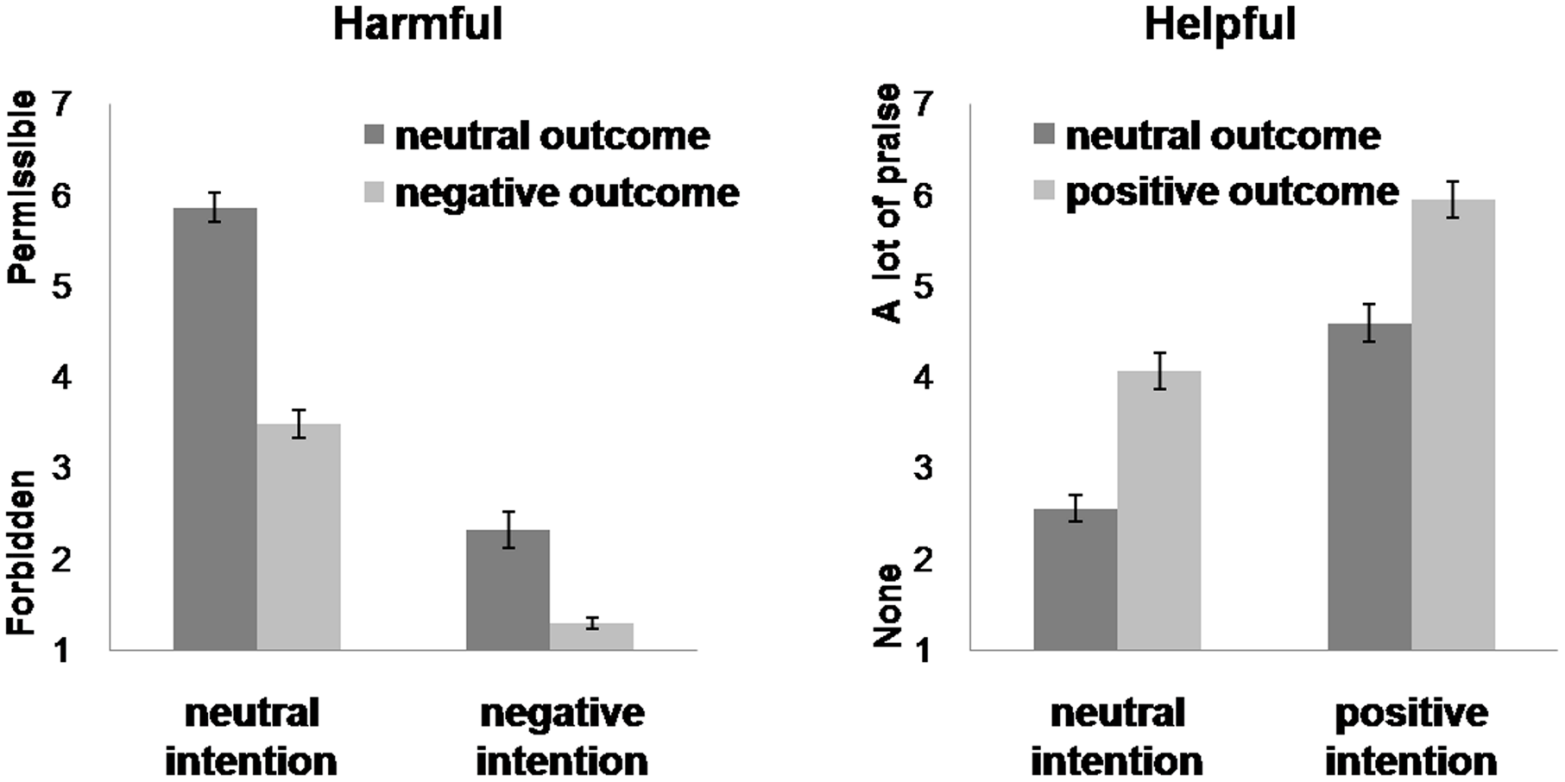
**Moral permissibility **(left)** and praise **(right)** judgments.** Error bars represent standard error.

For the values of permissibility, the main effects of intention and outcome were both significant. Agents with negative intentions were judged more non-permissible than those with neutral intentions [negative, 1.81 ± 0.09; neutral, 4.67 ± 0.17; *F*(1,26) = 216.12, *p <* 0.001, partial η^2^ = 0.89]. Agents producing negative outcomes were judged more non-permissible than those causing neutral outcomes [negative, 2.38 ± 0.11; neutral, 4.09 ± 0.12; *F*(1,26) = 78.54, *p <* 0.001, partial η^2^ = 0.89]. The interaction between intention and outcome was significant [*F*(1,26) = 129.56, *p <* 0.001, partial η^2^ = 0.83]. *Post hoc* tests showed that the difference between no harm and accidental harm was larger than that between attempted harm and successful harm (no harm, 5.86 ± 0.16; accidental, 3.48 ± 0.20; attempted, 2.32 ± 0.15; successful, 1.29 ± 0.06) (**Figure 2**).

#### Moral Judgments of Help Scenarios

Predicted main effects of intention and outcome were observed. Agents with positive intentions were judged more praiseworthy than agents with neutral intentions [positive, 5.27 ± 0.18; neutral, 3.31 ± 0.16, *F*(1,27) = 149.15, *p <* 0.001, partial η^2^ = 0.85]. Agents producing positive outcomes were judged more praiseworthy than those causing neutral outcomes [positive, 5.01 ± 0.17; neutral, 3.57 ± 0.15, *F*(1,27) = 163.37, *p <* 0.001, partial η^2^ = 0.86]. However, there was no significant interaction between intention and outcome [*F*(1,27) = 0.44, *p* = 0.514, partial η^2^ = 0.02] (**Figure 2**).

#### Reaction Time of Harm Scenarios

The main effect of intention was significant [*F*(1,26) = 23.83, *p <* 0.001, partial η^2^ = 0.48]. Judgments of negative intentions were faster than of neutral intentions (negative, 1628 ± 117 ms; neutral, 1347 ± 89 ms). The interaction between intention and outcome was also significant [*F*(1,26) = 41.58, *p <* 0.001, partial η^2^ = 0.62]. *Post hoc* tests showed that judgments of neutral outcomes were longer than that of negative outcomes when the intention of the agent was negative (negative, 1516 ± 110 ms; neutral, 1177 ± 74 ms). However, when the agent had neutral intention, the reaction times of neutral outcomes were significantly faster than that of negative outcomes (neutral, 1431 ± 104 ms; negative, 1824 ± 136 ms).

#### Reaction Time of Help Scenarios

There was a main effect of outcome [*F*(1,27) = 29.49, *p <* 0.001, partial η^2^ = 0.52] and a significant interaction between intention and outcome [*F*(1,27) = 25.98, *p <* 0.001, partial η^2^ = 0.49]. *Post hoc* tests showed that only when the agents had positive intention, the judgments were faster for positive outcomes (1281 ± 100 ms) than neutral outcomes (1799 ± 135 ms). When the agents had neutral intention, no difference was found in reaction times between positive outcomes and neutral outcomes.

### ERP Results

#### ERP Components and Analysis

**FIGURE 3:**
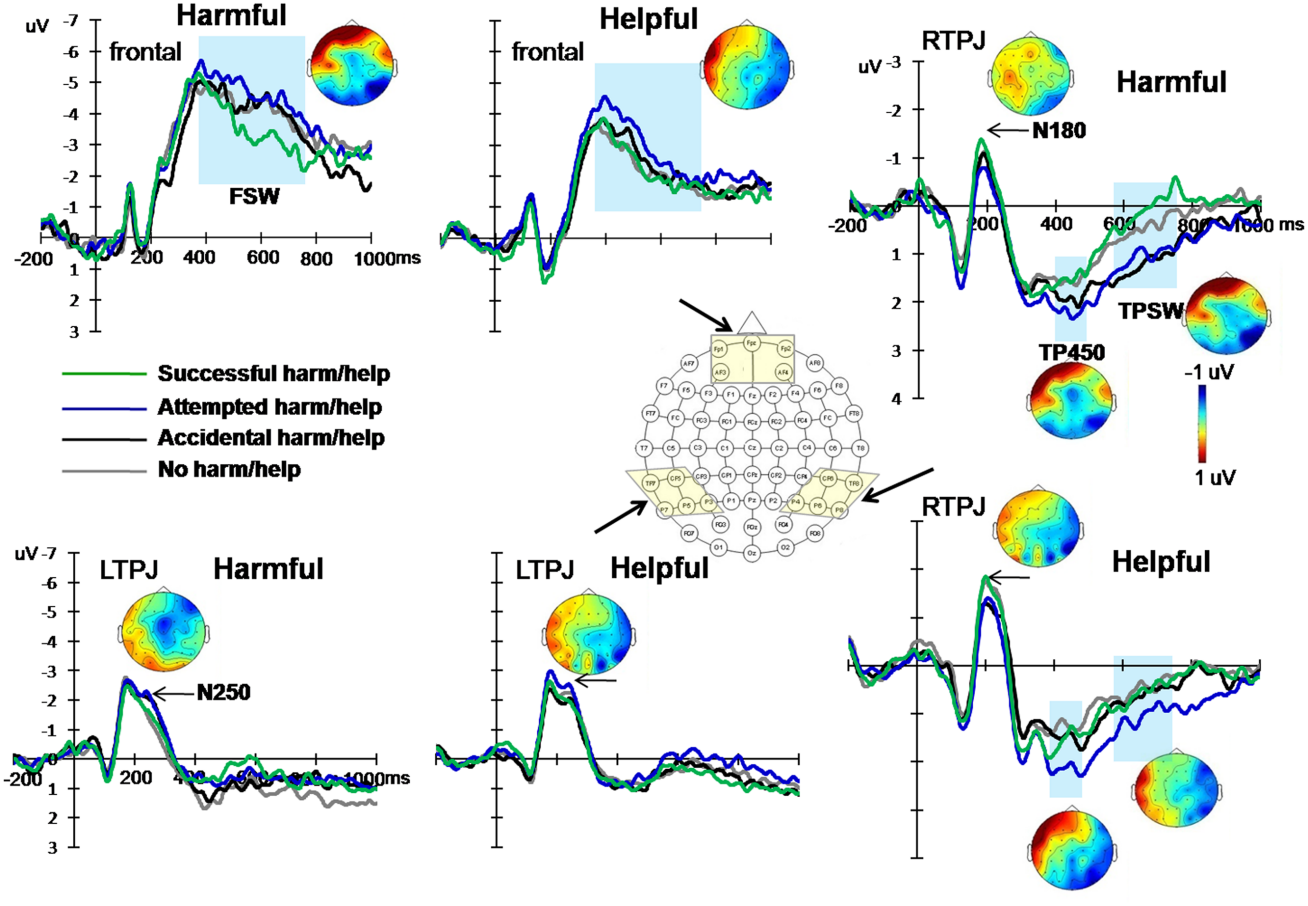
**Grand-averaged ERPs to the same keywords of successful action (green line), attempted failed action (blue line), accidental action (black line) and neutral action (gray line) conditions over frontal, left and right temporal-parietal areas, from two experiments.** Shown next to the waveforms are the associated scalp distributions of the five differential effects during the 160–210 ms (N180), 230–270 ms (N250), 380—780 ms (FSW), 400–500 ms (TP450), and 580–780 ms (TPSW) time windows for the difference waves between attempted and successful actions (subtracting ERPs of attempted conditions from successful conditions).

The grand averaged ERP patterns of harmful and helpful experiments were similar (see Supplementary Materials). Following the methodology of previous studies and after examination of the grand average ERPs, the ERP components at three areas were selected: frontal area (average amplitudes of electrodes FPZ, FP1, FP2, AF3, and AF4), left temporo-parietal area (average amplitudes of electrodes CP5, P3, P5, P7, and TP7) and right temporo-parietal area (average amplitudes of electrodes CP6, P4, P6, P8, and TP8). Based on the hypothesis and visual inspection, two early components and three late waves were analyzed: two early negative components peaking from 160 to 210 ms (N180), from 230 to 270 ms (N250) over bilateral temporo-parital areas; a positive component peaking between 400 and 500 ms (TP450) and a late slow-wave from 580 to 780 ms (TPSW) over bilateral temporo-parital areas; and a frontal slow-wave from 380 to 780 ms (FSW) recorded over frontal area. **Figure 3** presents the mean ERPs over frontal and bilateral temporal-parietal areas during four experimental conditions.

**FIGURE 4:**
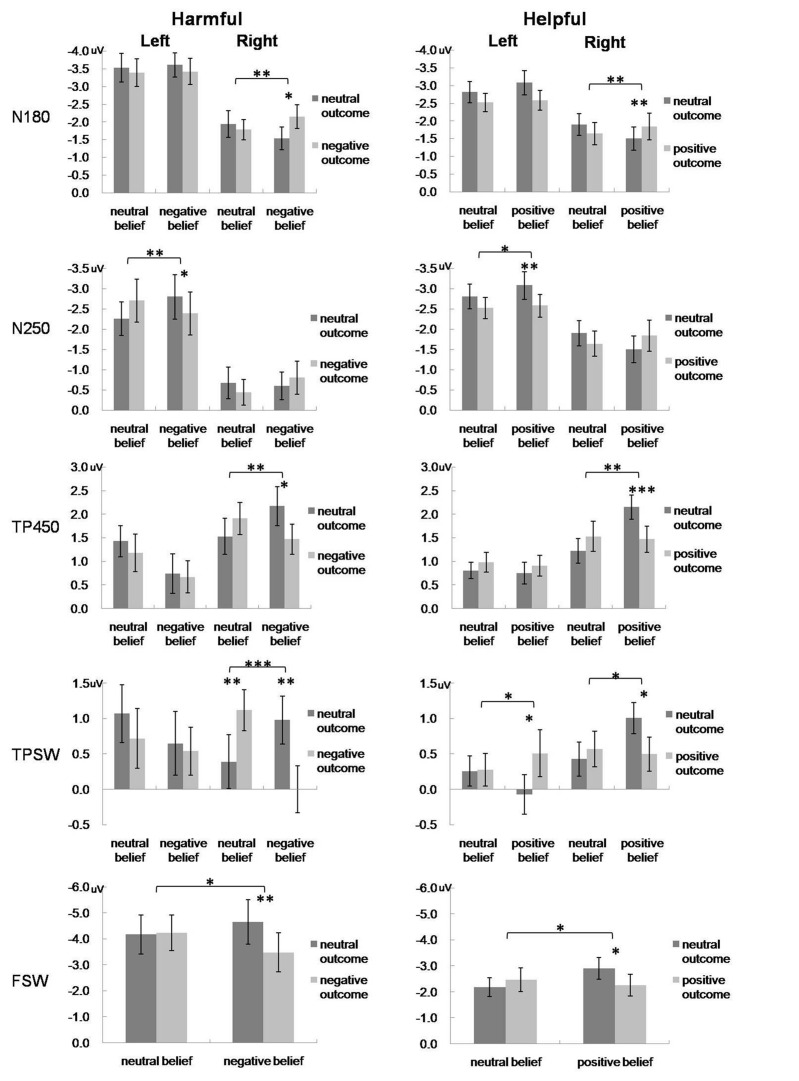
**Mean amplitudes of the N180, N250, TP450 and TPSW recorded from selected left and right TPJ electrodes, and mean amplitudes of the FSW recorded from selected prefrontal electrodes for the four experimental conditions in harmful **(left)** and helpful **(right)** moral judgments.** Error bars represent standard error. ^∗^*p* < 0.05; ^∗∗^*p* < 0.01; ^∗∗∗^*p* < 0.001.

For the statistical analysis, we analyzed the differences in ERP waveforms recorded during the four experimental conditions in both experiments. At left and right temporo-parietal areas, the peak amplitudes of N180 and N250 and the mean amplitudes of TP450 and TPSW were computed. At the frontal area, the mean amplitude of FSW was computed. For the temporo-parietal ERP components, amplitudes of each component were measured by a three-way repeated-measures analysis of variance (ANOVA) of 2 (intention: valenced, neutral) × 2 (outcome: valenced, neutral) × 2 (hemisphere: left, right). For the FSW, a two-way repeated-measures ANOVA of 2 (intention: valenced, neutral) × 2 (outcome: valenced, neutral) was conducted. The Greenhouse–Geisser correction was applied to adjust the degrees of freedom of the *F* ratios. The statistical results and effects are listed in **Table 1** and **Figure 4.**

**Table 1:** Mean amplitudes and 95% confidence intervals for ERP components in harm/help scenarios (μV).

Harm/Help	ERP	Left/Right	Successful *M* (95% CI)	Attempted *M* (95% CI)	*p*	Accidental *M* (95% CI)	All-neutral *M* (95% CI)	*p*
Harm	N180	L	-3.42 (-4.19,-2.66)	-3.61 (-4.31,-2.91)	0.49	-3.39 (-4.20,-2.58)	-3.53 (-4.36,-2.70)	0.52
		R	-2.15 (-2.82,-1.48)	-1.54 (-2.20,-0.88)	0.04^∗^	-1.78 (-2.36,-1.19)	-1.94 (-2.73,-1.15)	0.49
	N250	L	-2.39 (-3.48,-1.30)	-2.80 (-3.92,-1.67)	0.03^∗^	-2.71 (-3.80,-1.63)	-2.26 (-3.11,-1.42)	0.08
		R	-0.80 (-1.64,0.04)	-0.60 (-1.29,0.10)	0.44	-0.44 (-1.09,0.21)	-0.67 (-1.47,0.12)	0.26
	TP450	L	0.67 (-0.03,1.37)	0.74 (-0.11,1.60)	0.79	1.18 (0.35,2.00)	1.43 (0.75,2.11)	0.38
		R	1.47 (0.81,2.14)	2.17 (1.33,3.02)	0.03^∗^	1.91 (1.21,2.61)	1.53 (0.74,2.31)	0.11
	TPSW	L	0.54 (-0.17,1.24)	0.65 (-0.27,1.57)	0.73	0.72 (-0.15,1.58)	1.07 (0.24,1.90)	0.22
		R	0.00 (-0.67,0.68)	0.98 (0.29,1.68)	0.00^∗∗^	1.12 (0.53,1.71)	0.39 (-0.38,1.16)	0.01^∗∗^
	FSW	/	-3.48 (-5.03,-1.93)	-4.65 (-6.41,-2.88)	0.04^∗^	-4.24 (-5.65,-2.83)	-4.17 (-5.72,-2.61)	0.84
Help	N180	L	-3.40 (-3.94,-2.86)	-3.72 (-4.36,-3.09)	0.10	-3.08 (-3.51,-2.66)	-3.37 (-3.91,-2.84)	0.08
		R	-2.41 (-3.08,-1.73)	-1.94 (-2.58,-1.30)	0.00^∗∗^	-1.97 (-2.54,-1.40)	-2.28 (-2.83,-1.74)	0.16
	N250	L	-2.58 (-3.15,-2.01)	-3.08 (-3.76,-2.39)	0.00^∗∗^	-2.52 (-3.06,-1.98)	-2.81 (-3.43,-2.19)	0.18
		R	-1.84 (-2.60,-1.07)	-1.50 (-2.19,-0.82)	0.11	-1.64 (-2.27,-1.00)	-1.90 (-2.53,-1.27)	0.19
	TP450	L	0.91 (0.46,1.37)	0.75 (0.28,1.21)	0.40	0.98 (0.55,1.40)	0.81 (0.45,1.16)	0.29
		R	1.47 (0.90,2.04)	2.15 (1.63,2.68)	0.00^∗∗∗^	1.53 (0.88,2.19)	1.22 (0.68,1.76)	0.19
	TPSW	L	0.51 (-0.16,1.17)	-0.07 (-0.65,0.50)	0.03^∗^	0.28 (-0.19,0.76)	0.26 (-0.18,0.70)	0.91
		R	0.50 (0.01,0.99)	1.01 (0.55,1.46)	0.01^∗^	0.57 (0.05,1.09)	0.43 (-0.06,0.91)	0.54
	FSW	/	-2.26 (-3.13,-1.39)	-2.90 (-3.75,-2.04)	0.04^∗^	-2.47 (-3.42,-1.52)	-2.19 (-2.93,-1.45)	0.33


*L, left; R, right; *M*, mean amplitudes; ^∗^*p* < 0.01; ^∗∗^*p* < 0.05; ^∗∗∗^*p* < 0.001; CI, confidence interval.*

#### ERP Component Effects

##### Early ERP effects over TPJ area for harm scenarios

For the N180, the repeated-measures ANOVA only revealed a three-way interaction [*F*(1,26) = 4.42, *p* = 0.045, partial η^2^ = 0.15]. *Post hoc* tests showed that only in the right hemisphere, the N180 was more negative for successful harm than attempted harm.

For the N250, the main effect of hemisphere was significant [*F*(1,26) = 15.25, *p* = 0.001, partial η^2^ = 0.37], with that the N250 amplitude was more negative in the left hemisphere than the right hemisphere. The three-way interaction was also significant [*F*(1,26) = 9.65, *p* = 0.005, partial η^2^ = 0.27]. *Post-hoc* tests showed that only in the left hemisphere, the N250 was more negative for attempted harm than successful harm.

##### Early ERP effects over TPJ area for help scenarios

For the N180, the interaction between outcome and hemisphere was significant [*F*(1,27) = 4.67, *p* = 0.04, partial η^2^ = 0.15]. Most importantly, there was a significant three-way interaction [*F*(1,27) = 7.95, *p* = 0.009, partial η^2^ = 0.23]. *Post hoc* tests showed that only in the right hemisphere, the N180 was more negative for successful help than attempted help.

For the N250, the main effects of outcome and hemisphere were both significant. The N250 was more negative for neutral outcome than positive outcome [*F*(1,27) = 5.27, *p* = 0.03, partial η^2^ = 0.16] and was more negative in the left hemisphere than the right hemisphere [*F*(1,27) = 9.075, *p* = 0.006, partial η^2^ = 0.25]. The interaction between outcome and hemisphere was significant [*F*(1,27) = 5.142, *p* = 0.032, partial η^2^ = 0.16], such that in the left hemisphere, the N250 was more negative for neutral outcome than positive outcome. The three-way interaction was also significant, *F*(1, 27) = 5.83, *p* = 0.023, partial η^2^ = 0.18. Again, only in the left hemisphere, the N250 was more negative for attempted help than successful help.

##### Late ERP effects over TPJ area for harm scenarios

For the TP450, there was a significant interaction between intention and hemisphere [*F*(1,26) = 5.04, *p* = 0.033, partial η^2^ = 0.16], whereby amplitudes were significantly larger for neutral compared with negative trials only in the left hemisphere. Most importantly, TP450 amplitudes exhibited a three-way interaction [*F*(1,26) = 5.47, *p* = 0.027, partial η^2^ = 0.17]. *Post hoc* tests showed that only in the right hemisphere, the TP450 was larger for attempted harm than successful harm.

For the TPSW, the main effect of intention was significant [*F*(1,26) = 5.34, *p* = 0.029, partial η^2^ = 0.17], indicating that the TPSW amplitude was more positive for the neutral intention than the negative intention. The interaction between intention and outcome was significant [*F*(1,26) = 8.79, *p* = 0.006, partial η^2^ = 0.25], indicating that the TPSW amplitude was more positive for attempted harm than successful harm. Most importantly, the three-way interaction was significant [*F*(1,26) = 15.97, *p <* 0.001, partial η^2^ = 0.38]. *Post hoc* tests showed that only in the right hemisphere, the TPSW amplitude was more positive for attempted harm than successful harm and was more positive for accidental harm than no harm (accidental, 1.12 ± 0.29 μV; no harm, 0.39 ± 0.38 μV).

##### Late ERP effects over TPJ area for help scenarios

For the TP450, there was a significant main effect of hemisphere, with the TP450 more positive in the right hemisphere than the left hemisphere [*F*(1,27) = 5.88, *p* = 0.022, partial η^2^ = 0.18]. The interactions of outcome × hemisphere [*F*(1,27) = 4.43, *p* = 0.045, partial η^2^ = 0.14] and intention × outcome [*F*(1,27) = 5.75, *p* = 0.024, partial η^2^ = 0.18] were both significant. Most importantly, the TP450 amplitudes exhibited a three-way interaction, *F*(1, 27) = 5.81 *p* = 0.023, partial η^2^ = 0.18. *Post hoc* tests showed that only in the right hemisphere, the TP450 was larger for attempted help than successful harm.

For the TPSW, the interaction between outcome and hemisphere was significant, *F*(1,27) = 6.47, *p* = 0.017, partial η^2^ = 0.19. Most importantly, the three-way interaction was also significant, *F*(1,27) = 6.98, *p* = 0.014, partial η^2^ = 0.21. *Post hoc* tests showed that in the left hemisphere, the TPSW was more positive for successful help than attempted help. In contrast, in the right hemisphere, the TPSW was more positive for attempted help than successful help.

##### Late ERP effects over frontal area for harm scenarios

For the FSW, there was a significant interaction of intention × outcome [*F*(1,26) = 4.98, *p* = 0.035, partial η^2^ = 0.16]. *Post hoc* tests showed that the FSW amplitude was more negative for attempted harm than successful harm.

##### Late ERP effects over frontal area for help scenarios

Similar to the harmful experiment, there was a significant interaction of intention × outcome of the FSW, *F*(1,27) = 6.86, *p* = 0.014, partial η^2^ = 0.20. *Post hoc* tests showed that the FSW was more negative for attempted help than successful help.

## Discussion

One aim of the present study was to examine ERP responses to keywords integrating the information of harmful/helpful intention and outcome in moral judgment. The ERPs were time-locked to the keywords of intention. In each trial, the foreshadow information actually have implied the possible outcome of the agent’s action. So, when the keyword of intention was presented, subjects not just decoded but also integrated the intention with outcome information. This integration processing has lead to the significant interaction effect between intention and outcome in previous studies (Young et al., 2007, 2011; Young and Saxe, 2008) and in the present study. Consistent with our hypothesis, the ERPs showed differences between conditions in early and late time windows over prefrontal and temporo-parietal electrodes. We presume that these frontal and bilateral temporo-parietal effects reflect, at least in part, the participants’ online processing of the integration of intention and outcome information to make moral judgments.

During early time windows, two negative ERP components, N180 and N250, over bilateral temporo-parietal areas were found. These components differentiated between successful and attempted conditions in both experiments. Because the keywords of intention in these two conditions were the same, differences in ERP activities cannot be attributed to physical differences between stimuli but rather to the mental processes. During the time window from 160 to 210 ms, the successful harm/help conditions induced more negative N180 than attempted conditions. This early effect was consistent with the fast automatic reaction to moral valence information found in previous studies (Van Berkum et al., 2009; Decety and Cacioppo, 2012; Yoder and Decety, 2014; Gui et al., 2015). Yoder and Decety (2014) reported the amplitude differences on N1 component between morally good and bad actions. In our previous study, we found that the N1 amplitudes elicited by moral pictures were significantly more negative than those elicited by non-moral pictures and suggested that this early ERP effect reflected the moral intuition processing without the emotional impact (Gui et al., 2015). By using high-density ERP techniques, Decety and Cacioppo (2012) found that people could distinguish between intentional and accidental harm in as fast as 62 ms post-stimulus. Van Berkum et al. (2009) reported a rapid ERP response to the first word that indicates a clash with the reader’s value system within 200 ms. According to the social intuitionist model (Haidt, 2001), when dealing with the information about morals, people would automatically give harsher evaluations to moral transgressors and more favorable evaluations to those who uphold moral standards. In the conditions of successful harm and help, the agent kills or saves a human life intentionally and successfully. Subjects may have a quick, automatic evaluations to give harsher punishment to violators and bigger reward to people who behave morally. This kind of rapid, automatic reaction may be reflected by the more negative N180 of successful conditions in both experiments. In other words, the condition of successful harm was more morally “bad” than the condition of attempted but failed harm, and the actions with higher moral valence induced more negative N180 amplitude. In the same way, the successful help condition induced larger N180 because this condition was more morally “good” than the attempted help condition. These effects only reached significance over right temporo-parietal areas, which were consistent with many previous findings: ERP studies have found significant activity in right TPJ during spontaneous trait inference and spontaneous goal inference processing (Van Duynslaeger et al., 2007; Van der Cruyssen et al., 2009). FMRI studies also reported that right TPJ contributes more to moral intuition than moral deliberation (Young and Saxe, 2009; Harenski et al., 2010). In a word, the effect of the N180 demonstrated a rapid response over right temporo-parietal electrodes to morally extreme information.

Over the left hemisphere, there was an early negative component peaking at 250 ms, which was named as N250. Different from the N180 effects, the N250 of attempted conditions were more negative than that of successful conditions. This effect was consistent with previous ERP findings: One ERP study showed that decoding mental states from pictures of eyes induced a negative component which started 270 ms post-stimulus (Sabbagh et al., 2004). Another study reported a larger amplitude of N250 over parietal electrodes for private intention compared to communicative and physical intentions and suggested that N250 was related to the early cognitive processing of intention information (Wang et al., 2012). Proverbio and Riva (2009), Proverbio et al. (2010) found that pictures of comprehensible behaviors induced larger posterior N250 compared to incomprehensible behaviors and suggested that this component reflected the recognition and comprehension processing of action intention. Besides, the N250 effect only reached significance over left temporo-parietal electrodes. Previous studies showed that left TPJ may be indexing differences in generic perspective processing during mentalizing (Perner et al., 2006; Young et al., 2011). Based on these results, we suggest the N250 effect might reflect the early representation and integration processing of moral intention and outcome information.

During late time windows, two late waves, TP450 and TPSW, over bilateral temporo-parietal electrodes and one slow wave, FSW, over frontal electrodes differentiated between conditions, especially between attempted and successful harm/help conditions. Over right temporo-parietal area, the positive component during the 400–500 ms time window (TP450) appeared and was exceptionally large for attempted harm/help conditions. This component has been demonstrated as an index of complex mental state processing in a previous TOM study and the activity of right TPJ could account for most of its variance (McCleery et al., 2011). In the present study, the significant interaction of intention × outcome on TP450 over right hemisphere might reflect the complex integration processing of intention and outcome information.

The TPSW component, in the later time window from 580 to 780 ms, not only distinguished between the attempted and successful harm/help conditions over right hemisphere, but also between no harm and accidental harm in Experiment 1 and between attempted help and successful help over left hemisphere in Experiment 2. Similar late positive components had been reported in many previous ERP studies, which were specifically associated with belief processing (Liu et al., 2009; Geangu et al., 2013). Over prefrontal area, consistent with previous studies, a slow wave from 380 to 780 ms (FSW) was found, which was larger in the attempted harm/help conditions than successful harm/help conditions. Frontal ERP effects have been found and were related to the mental states processing and inhibitory control in many previous studies (Chen et al., 2009; Liu et al., 2009; McCleery et al., 2011; Geangu et al., 2013). Here, our results suggested that the prefrontal activity started from 380 ms after stimulus presentation and the prefrontal effects were paralleled by similar findings at the temporo-parietal area, which have been specifically associated with differentiating and reasoning between conditions.

Another aim of this study was to explore the cognitive mechanism and neural temporal dynamics of integration of helpful intention and outcome. For the behavioral results, unlike in the harm context, we did not find the significant interaction between intention and outcome but only the main effects of these two factors. For the ERP results, in the help experiment, significant outcome × hemisphere interactive effects were found for N180, N250, TP450 and TPSW components, while these did not reach significance in the harm experiment. We suggest two possible reasons to explain these differences between helpful and harmful judgment. One reason is that in help scenarios, the importance of the outcome factor was greater, which neutralized the interactive effect between intention and outcome. Error management theory suggest that when people make a judgment (for instance, judge whether sticks are snakes or not), they can make either a false-positive error (inferring that it is a snake when it is actually not) or a false-negative error (inferring that it is not a snake when it actually is). People more often make the false-positive error because the cost of this kind of error is less over evolutionary time (Haselton and Buss, 2000; Haselton and Nettle, 2006). In the attempted and accidental help conditions of the present study, agents in the described stories either have positive intents to help others, or their actions have lead to positive consequences. According to this theory, although agents in these two conditions have made false-positive errors, subjects still gave them more favorable evaluations, which may neurtralize the interaction of intention and outcome in helpful condition. Another reason is the differences between the tasks. In the help contexts, we asked participants to judge how much moral praise the agent deserves, but in the harm condition, the task was to judge the moral permissibility of the agent’s action. Different question types could induce different psychological processing for moral judgments (Christensen and Gomila, 2012). When participants were asked for the permissibility of a moral transgression, they appeared to base primarily on intention information. However, for the assignment of praise and blame, both mental states and the causal link between the agent’s actions and the harmful consequences is important (Cushman, 2008). A similar asymmetry between moral judgment of good and bad has been reported in a previous study: for the negative impulsive actions elicited a discounting of moral blame, but the positive impulsive actions did not elicit a discounting of moral praise (Pizarro et al., 2003).

To the best of our knowledge, this is the first study using ERPs to assess the temporal dynamics of integration processing of intention and outcome in harmful and helpful moral judgment contexts. The findings suggest that the neural mechanism of the integration processing of moral intention and outcome may involve three stages. The first stage involves a fast response over right temporo-parietal area, reflected in our finding that successful harm/help conditions induced more negative N180 than attempted harm/help conditions. The second stage involves representation of intention information and early integration processing over left temporo-parietal area, indicated by the larger N250 for attempted harm/help conditions. The third stage involves late integration and reasoning processing over prefrontal and temporo-parietal areas, reflected by the TP450, TPSW and FSW effects.

There are some limitations of the present study. First, when the intention keyword was presented, before completing the integration process, participants might have encoded the intention information first. Future studies should manipulate the sequence of intention and outcome presentation to clearly separate these two different processing phases. Second, as the stories in harmful moral scenarios were adopted from previous fMRI studies (Young et al., 2007, 2010, 2011), the background information in some stories might give readers a clue about the tendency of the agent’s intention before the intention keyword was presented to them (e.g., Because the white powder is labeled “sugar,” Grace believes that it is “safe”). However, we have found similar patterns of ERP components in judgments about helpful moral scenarios when we keep the information constant across different conditions of the same scenarios, so the significant differences in early N180 component between conditions with the same moral keywords might not be attributed to those intention clue differences in background information. Anyway, further studies are still required to obtain a purer intention component when investigating related questions. Finally, the present study demonstrated that prefrontal and temporo-parietal activities could reflect the processing of the integration of intention and outcome. However, the spatial resolution of ERP technique is not high enough. Future studies should use high-density electrical techniques combined with fMRI to identify the source of these ERP components more accurately, which would be a meaningful contribution to the understanding of the neural mechanisms of moral judgment.

## Conclusion

Our findings suggested a possible time course of neural activation during integration of moral intention and outcome, starting from the right temporo-parietal area, more negative N180 of successful harm/help conditions reflected a rapid intuitionist response in the earliest time window. Then the larger N250 for attempted harm/help over left temporo-parietal area implied the representation and early integration processing. The late ERP effects (FSW, TP450 and TPSW) implied the integration and reasoning processing over frontal and bilateral temporo-parietal areas in late time window. These results highlighted the critical role of neural activation over right temporo-parietal areas in both early automatic responses to moral actions and late moral integration processing.

## Conflict of Interest Statement

The authors declare that the research was conducted in the absence of any commercial or financial relationships that could be construed as a potential conflict of interest.
