# Supplementary material for: Temporal Dynamics of the Integration of Intention and Outcome in Harmful and Helpful Moral Judgment
Source: Front Psychol. 2016 Jan 11;6:2022. doi: 10.3389/fpsyg.2015.02022 (PMC4708004; doi:10.3389/fpsyg.2015.02022)
Supplement: Supplementary file 1 [file Presentation_1.PDF]

Supplementary materials

Figure 1. Grand-averaged ERPs elicited in successful harm (green line), attempted failed harm (blue line), accidental harm (red line) and neutral (black line) conditions for channels over frontal and bilateral temporo-parietal areas in harmful experiment.

Figure 2. Grand-averaged ERPs elicited in successful help (green line), attempted failed help (blue line), accidental help (red line) and neutral (black line) conditions for channels over frontal and bilateral temporo-parietal areas in helpful experiment.

Figure 3. Grand-averaged ERPs to the same key words of successful harm (green line), attempted failed harm (blue line), accidental harm (black line) and neutral harm (grey line) conditions at right posterior electrode (P6), left posterior electrode (P7) and prefrontal electrode (FPZ). Left pannel was the ERPs of all scenarios in harmful experiment, right pannel was the ERPs of 21 scenarios that with the identical sentence before intention keyword.
