# Supplementary material for: Temporal Dynamics of the Integration of Intention and Outcome in Harmful and Helpful Moral Judgment
Source: Front Psychol. 2016 Jan 11;6:2022. doi: 10.3389/fpsyg.2015.02022 (PMC4708004; doi:10.3389/fpsyg.2015.02022)
Supplement: Supplementary file 2 [file Presentation_2.PDF]

Supporting Information

Harmful scenarios:

Coffee.

Grace and her friend are taking a tour of a chemical plant. When Grace goes over to the coffee machine to pour some coffee, Grace's friend asks for some sugar in hers. There is white powder in a container by the coffee.

The white powder is just the regular sugar that the scientists use each day, and is therefore perfectly safe to have in coffee.

The white powder is a very toxic substance left behind by a scientist, and is therefore deadly when ingested in any form.

The container is labeled "sugar," so Grace believes that the white is **safe**.

The container is labeled "toxic," so Grace believes that the white powder is **dangerous**.

Grace puts the substance in her friend's coffee. Her friend drinks the coffee and is fine.

Grace puts the substance in her friend's coffee. Her friend drinks the coffee and dies.

Jellyfish.

Janet and her neighbor are kayaking in a part of the ocean with lots of jellyfish. Janet's neighbor asks her if she should go for a swim.

Because the jellyfish do not sting and are totally harmless, swimming in the ocean is safe.

Because the jellyfish sting and their stings are fatal, swimming in the ocean is dangerous.

Janet believes the ocean's jellyfish is **harmless**.

Janet believes the ocean's jellyfish is **dangerous**.

Janet tells her neighbor to go for a swim. Her neighbor does, and is fine.

Janet tells her neighbor to go for a swim. Her neighbor does, and dies.

Bridge.

Ray and his girlfriend are hiking. They come across a long narrow bridge that spans a steep canyon.

The bridge happens to be extremely sturdy and can easily carry the weight of many people at once.

The bridge happens to be extremely unsteady and cannot carry the weight of even one very light person.

Ray believes that walking on the bridge is **safe**.

Ray believes that walking on the bridge is **dangerous**.

Ray says nothing as his girlfriend starts walking across the bridge. His girlfriend is fine.

Ray says nothing as his girlfriend starts walking across the bridge. His girlfriend dies.

Mushrooms.

Lauren and an acquaintance are camping in the woods. Lauren spots some wild mushrooms growing along the campsite. Lauren studies the mushrooms and consults her plant life guide.

The mushrooms happen to be edible.

The mushrooms happen to be lethal.

Lauren believes eating these mushrooms is **safe**.

Lauren believes eating these mushrooms is **dangerous**.
Lauren offers the mushrooms to her acquaintance. Her acquaintance eats them and is fine.
Lauren offers the mushrooms to her acquaintance. Her acquaintance eats them and dies.

Motorboat.
George is driving his motorboat in the bay on his way home. He spots a swimmer in the far distance.
The swimmer is, in fact, fine and is waving at George for fun as he does with people in the bay.
The swimmer is, in fact, drowning and is waving frantically at George for help.
George believes that the swimmer is **safe**.
George believes that the swimmer is **in danger**.
George drives home, leaving the swimmer behind. The swimmer is fine.
George drives home, leaving the swimmer behind. The swimmer dies.

Vitamin.
Gil is instructed by a doctor to give his senile wife pills for her heart disease. The doctor says that she must not intake vitamin K within an hour to take the pills safely. One day, his wife tries a new kind of fruit.
The new kind of fruit does not have vitamin K, so it is safe for Gil's wife to take the pills right away.
The new kind of fruit is high in vitamin K, so it is deadly for Gil's wife to take the pills right away.
Gil does his research and believes that giving his wife the pills is **safe**.
Gil does his research and believes that giving his wife the pills is **dangerous**.
Gil gives his wife the pills right away. His wife is fine.
Gil gives his wife the pills right away. His wife dies.

Laboratory.
Dan is giving a visitor a tour of a laboratory. Before visitors enter the testing room, all test tubes containing disease antigens must be contained in a chamber by flipping a switch. A repairman has just come to fix the switch, which had been broken.
The switch has been successfully repaired, so the test tubes are quite safely contained. Thus, anybody who enters the room will be safe.
The switch is still broken, so the test tubes are out in the open. Thus, anybody who enters the room will be dangerous.
Dan believes the switch has been **repaired**.
Dan believes the switch is still **broken**.
Dan tells the visitor to enter the testing room. The visitor is fine.
Dan tells the visitor to enter the testing room. The visitor dies.

Sesame.
Kate is a waitress preparing to take a meal out to a customer's table. The customer is with his friends, and he orders a meal that calls for sesame seeds.
The customer happens to love sesame seeds and will have no problem at all if he eats the sesame

seeds in his meal.
The customer happens to be highly allergic to sesame seeds and will most likely die if he eats them in his meal.
After overhearing part of the customer’s conversation with his friends, Kate believes that the customer eating the sesame seeds is **dangerous**.
After overhearing part of the customer’s conversation with his friends, Kate believes that the customer eating the sesame seeds is **safe**.
Kate puts the sesame seeds in. The customer enjoys his meal and is fine.
Kate puts the sesame seeds in. The customer eats his meal and dies.

Biking.
Ginny’s bike has just come back from the repair shop. The brakes had not been working.
The brakes on Ginny’s bike are working perfectly now, so the bike is safe to ride.
The brakes on Ginny’s bike still are not working at all, so the bike is very dangerous to ride.
The people at the repair shop told Ginny that the brakes are still broken and gave her a demonstration to explain why they are not fixed, so she believes the bike is **dangerous**.
The people at the repair shop told Ginny that the brakes are fully fixed now and gave her a demonstration to explain how they were fixed, so she believes the bike is **safe**.
Ginny lends the bike to her classmate. Her classmate is fine.
Ginny lends the bike to her classmate. Her classmate dies.

Iron.
Josephine and her little sister are in the bathroom doing makeovers by the sink. Josephine had straightened her hair earlier in the day using a straightening iron. The iron is still on the sink.
Josephine’s mother had turned the button on the iron off 3 h ago, so it is no longer hot and is perfectly safe to touch.
Josephine’s mother had just used the iron herself 5 min ago, so it is still extremely hot and could cause severe burns.
Because the cord on the iron is still in the socket, Josephine believes that the iron is **dangerous**.
Because the cord on the iron was unplugged, Josephine believes that the iron is **safe**.
Josephine lets her sister continue to play by the sink. Her sister’s arm hits the iron and is fine.
Josephine lets her sister continue to play by the sink. Her sister’s arm hits the iron and she gets burned.

Meatloaf.
Rebecca works at a day care program. For lunch, Rebecca plans to make meatloaf for all the children. She opens a package of ground beef to make the meatloaf.
The meat is actually completely fresh, because the package was tightly sealed while stored in the refrigerator; thus, the meat is safe to eat.
The meat has some invisible but very deadly bacteria on it because of a small tear in the seal; thus, the meat is not safe to eat.
Because the expiration date on it passed 2 weeks ago, Rebecca believes that eating these beef are **dangerous**.
Because the expiration date on it is 2 weeks away from now, Rebecca believes that eating these

beef are **safe**.
Rebecca makes meatloaf out of the ground beef and serves it to the children. The children eat the meal and is fine.
Rebecca makes meatloaf out of the ground beef and serves it to the children. The children eat the meal and is poisoned.

River.
Jacob and his friend are rafting down the Colorado River. Jacob's friend is a novice. They stop by the bank to have a swim. Jacob's friend starts to swim downstream.
The next segment of the river is very tame this year. It is very easy to swim through, and the scenery is particularly nice.
The next segment of the river is very rough and fast this year. It is full of gigantic boulders that make it very dangerous to swim through.
Because Jacob has rafted down this part of the Colorado River before, he believes swimming in the river is **dangerous**.
Because Jacob has rafted down this part of the Colorado River before, he believes swimming in the river is **safe**.
Jacob silently watches as his friend swims downstream. His friend is fine.
Jacob silently watches as his friend swims downstream. His friend dies.

Logan Airport.
Andrew works at Logan Airport. He is in charge of ensuring that the runways are clear of debris that could damage planes during takeoff. A plane is about to take off on a distant runway.
The distant runway has been completely cleared. It is ready for the plane to use for takeoff.
The distant runway has not yet been cleared. There is potentially damaging debris on it.
Andrew checks with his supervisor and believes that taxing in the runway is **safe**.
Andrew checks with his supervisor and believes that taxing in the runway is **dangerous**.
Andrew gives the "OK" for the takeoff. The plane takes off and is fine.
Andrew gives the "OK" for the takeoff. The plane takes off but later failed and three passengers are killed.

Tracks.
Tyler works for a company that builds railway tracks for passenger trains. One day, Tyler's job is to fasten the track rods with an extra spike at dangerous corners.
The night team working the previous night was very efficient, and they have already added extra spikes to all corners in the first segment of the track. The whole segment is entirely safe.
The night team working the previous night was running late, so they did not finish adding extra spikes to all corners in the first segment of track. In some places, the track is still dangerous.
Tyler checks the work logs of the night team and believes that part of the track is **safe**.
Tyler checks the work logs of the night team and believes that part of the track is **dangerous**.
Tyler skips the whole first segment of track. The railway track is opened for commercial use, and passenger trains are fine.
Tyler skips the whole first segment of track. The railway track is opened for commercial use, and a passenger train derails and crashes, many passengers die.

Harness.

Stacey and her friend are going rock climbing, and they are going to use harnesses to scale a gigantic cliff. Stacey's friend starts to put on the new harness.

The new harness is a top-of-the-line model, in fine working condition, and completely safe to use.

The clamp on the new harness is actually subtly flawed, so the whole harness is incredibly unsafe to use.

Because Stacey bought the harness from a quality sports store, Stacey believes that using the harness is **safe**.

Because the clamp on the harness does not audibly click into place, Stacey believes that using the harness is **dangerous**.

Stacey straps her friend into the harness. Her friend is fine.

Stacey straps her friend into the harness. Her friend dies.

Igloo.

Sarah built a huge play igloo out of snowballs yesterday. Her friend is over today, and she is totally intrigued by the igloo.

The igloo is sturdily built. The snowballs have frozen solid into strong walls, so it is fine for Sarah's friend to play inside all shewants.

The snowballs in the igloo walls are not properly balanced and will cave in at moderate movement, so it is not safe for Sarah's friend to go inside.

Because she has built igloos like this many times before, Sarah believes that playing in the igloo is **safe**.

Because her mother warned her about playing in igloos like this, Sarah believes that playing in the igloo is **dangerous**.

Sarah tells her friend to go inside the igloo. Her friend is fine.

Sarah tells her friend to go inside the igloo. Her friend is crashed by the icy snowballs.

Ham.

Noel and a friend are having lunch on Saturday. They are making sandwiches with stuff from Noel's refrigerator, when Noel's friend says she would prefer a ham sandwich.

The ham in the refrigerator is high quality and was purchased just a day ago. It is fresh and ready to be used in sandwiches.

The ham in the refrigerator was purchased a week ago and has since become slightly spoiled, so it is not safe to eat.

Because Noel's mom always shops for meats only on Fridays, Noel believes that eating the ham is **safe**.

Because Noel's mom usually shops for meats only on Sundays, Noel believes that eating the ham is **dangerous**.

Noel makes a ham sandwich for her friend. Her friend is fine.

Noel makes a ham sandwich for her friend. Her friend gets food poisoning.

Popcorn.

Matt is babysitting at his cousin's house. They are eating popcorn. Suddenly, they hear a loud

beeping noise coming from the kitchen. Matt’s cousin gets up to investigate.
The beeping noise is from a smoke detector responding to leftover smoke coming from the microwave in which Matt and his cousin burned popcorn. The situation in the kitchen is not dangerous.
The beeping noise is from a carbon monoxide detector in the kitchen. Deadly carbon monoxide is escaping from the furnace under the kitchen. The kitchen is now dangerous.
Because Matt just came out from the kitchen, he believes that going to the kitchen is **safe**.
Because Matt just came out from the kitchen, he believes that going to the kitchen is **dangerous**.
Matt watches quietly while his cousin goes into the kitchen. His cousin is fine.
Matt watches quietly while his cousin goes into the kitchen. His cousin dies.

Veterinarian.
Zach is a student working at a veterinarian’s office. His friend comes to visit him and sees cookies shaped like bones laying out in the worker’s lounge. His friend asks if he can eat one.
One of Zach’s coworkers had baked the cookies for her birthday and had put them out for others to enjoy. They are delicious and safe to eat.
One of Zach’s coworkers had put the cookies in the lounge by accident. They are used to put dogs to sleep, so they are poisonous to eat.
Because people normally put food in the workers’ lounge to share, Zach believes that the cookies are **safe**.
Because the shape of the cookies the veterinarian uses with special chemicals to put dogs to sleep, Zach believes that the cookies are **dangerous**.
Zach invites his friend to eat a dog bone-shaped cookie. His friend is fine.
Zach invites his friend to eat a dog bone-shaped cookie. His friend dies.

Chairlift.
Zoe is on winter break. She is running a chairlift at a ski lodge. She has just taken over after lunch break and is starting her afternoon shift. The first passenger is a child.
This afternoon, the chairlift is functioning perfectly normally and can safely carry passengers of any size up the side of the ski slope.
This afternoon, a problem with the electricity is making the old chairs bounce violently, making the chairlift dangerous for small passengers.
Zoe saw her coworker send a group of children off on the chairlift before lunch. So she believes that riding the chairlift is **safe**.
Zoe’s supervisor told her that the chairlift was malfunctioning before lunch. So she believes that riding the chairlift is **dangerous**.
Zoe starts the chairlift, and the child gets on. The child is fine.
Zoe starts the chairlift, and the child gets on. The child falls and dies.

Safety Town.
Sam is babysitting a preschool boy. His job is to watch the boy at Safety Town, a class designed to help children know what to do in a fire. Today, the children will go into a fake house full of smoke.
The boy has very strong lungs and is also used to second-hand smoke, so he will be comfortable

going into the Safety Town smoking house.
The boy has asthma, which makes his lungs close up around smoke, so he will not be able to breathe if he goes into the Safety Town smoking house.
Sam believesthat the boy participating at Safety Town is **safe**.
Sam believes that the boy participating at Safety Town is **in danger**.
Sam watches the boy go into the Safety Town smoking house. The boy is fine.
Sam watches the boy go into the Safety Town smoking house. The boy has a severe asthma attack inside.

Malaria Pond.
Peter is traveling in Africa with a friend. His friend sees a pond and wants to go wading in it because it is very hot. His friend begins to walk toward the pond.
The pond is a good place for tourists. It does not contain any disease-carrying organisms. The water is unusually clean, so it is safe to wade in.
Malarial mosquitoes actually live in the pond. A single bite is enough to create an infection, so the pond is unsafe to wade in.
Peter believes wading in the pond is **safe**.
Peter believes wading in the pond is **dangerous**.
Peter encourages his friend to wade in the pond. His friend is fine.
Peter encourages his friend to wade in the pond. His friend is bitten by several mosquitoes and contracts malaria.

Spinach.
Will is grocery shopping for his grandmother, who adores spinach. Recently, there had been incidents of Escherichia coli contamination of bagged spinach, leading to a recall of all bagged spinach.
Bagged spinach has been restocked at many markets. It is 100% safe to eat and no longer contaminated with E. coli.
Bagged spinach has been restocked at many markets. However, some inspections are not thorough, and contaminated batches are still being missed.
At the market, Will sees that bagged spinach is being carried again, Will believes that eating the spinach is **safe**.
At the market, Will sees that bagged spinach is being carried again, Will believes that eating the spinach is **dangerous**.
Will buys his grandmother bagged spinach. His grandmother cooks some for dinner that evening and is fine.
Will buys his grandmother bagged spinach. His grandmother cooks some for dinner that evening and is violently ill.

Alarm.
Bill is house-sitting for some neighbors over the Columbus Day weekend. He is stepping out the door, late for a lunch meeting with a friend, when the fire alarm goes off.
The alarm is old and defective and goes off for no reason during the day but does turn off by itself after several minutes. The alarm needs to be replaced.

The alarm is very accurate and detects dangerous levels of heat. The alarm was purchased to prevent fires caused by overheating in the basement.
Bill remembers the neighbors' saying something about their alarm. He believes that the house is **safe**.
Bill remembers the neighbors' saying something about their alarm. He believes that the house is **in danger**.
Bill leaves the house to meet his friend. While he is gone, the alarm turns itself off and the house is fine.
Bill leaves the house to meet his friend. While he is gone, the alarm keeps sounding and a fire starts in the house.

Zoo.
Ryan is at the zoo with his nephew. They are watching the dolphin show when the nephew complains that his stomach hurts.
Ryan's nephew is really fine. His stomach sometimes hurts when he eats too much junk food, as on that day, but he usually feels a lot better after an hour or so.
Ryan's nephew is really sick. After his recent operation, his doctors had warned that stomach pain could indicate really serious complications.
Because Ryan's nephew ate too much cotton candy and fried dough that afternoon. Ryan believes that his nephew is **safe**.
Because Ryan's nephew had a major operation weeks ago. Ryan believes that his nephew is **in danger**.
Ryan takes his nephew to see the monkeys next. His nephew starts feeling better in no time.
Ryan takes his nephew to see the monkeys next. His nephew blacks out because of severe internal bleeding.

Bar.
Kevin is at a bar one night when he sees his sister sitting at the next table. His sister is laughing and drinking and seems to be having a good time.
Kevin's sister is drinking her first drink and does not plan to drink anything else that night so that she can drive herself home safely.
Kevin's sister is drinking her fifth drink and plans on driving herself home even though she is drunk and will be dangerous on the road.
Kevin's sister is very responsible, so Kevin believes that his sister will be **safe**.
Kevin's sister loves to party hard, so Kevin believes that his sister will be **in danger**.
Kevin pays his bill and leaves the bar without talking to his sister. His sister decides to leave soon after and is fine.
Kevin pays his bill and leaves the bar without talking to his sister. His sister decides to leave soon after and snaps her spine because a traffic crash.

Sushi.
Mitch and his colleagues are at a new sushi restaurant close to their office. Mitch happens to know the owner of the restaurant through a mutual friend.
The restaurant owner takes great care to ensure the freshness of all the fish prepared sushi-style.

Everything exceeds health standards. The tuna is a specialty.
The restaurant owner has purchased some of his fish at cheap but disreputable fish markets to save money. Some batches of fish, usually the tuna, have parasites.
After hearing his friend rave about it. Mitch thinks that ordering the tuna is **safe**.
After hearing his friend complain about it. Mitch thinks that ordering the tuna is **unsafe**
Mitch recommends the tuna to his colleagues at the table. One of his colleagues orders the tuna and ends up finding it quite good.
Mitch recommends the tuna to his colleagues at the table. One of his colleagues orders the tuna and ends up getting a nasty strain of parasites.

Fumigation.
Brian is helping out his neighbor for the weekend. His neighbor is away, but her house is getting fumigated (with poisonous fumes) because of pests.
The neighbor's Great Aunt Sally is at her bridge partner's house. She will not be back until late afternoon of the following day when the house is free of noxious fumes.
The neighbor's Great Aunt Sally is upstairs in her attic room. She has fallen fast asleep and will not wake up easily, even when the fumigators start pumping in the fumes.
Brian thinks that his neighbor's Great Aunt Sally has gone to stay with her bridge partner, as she had planned on doing that earlier in the day. So Brain believes that fumigating the house is **safe**.
Brian thinks his neighbor's Great Aunt Sally is still upstairs in her attic room. So Brain believes that fumigating the house is **dangerous**.
Brian tells the fumigators to start their job. They fill the house with poisonous gas, which gets rid of the pests. The neighbor's Great Aunt Sally is fine.
Brian tells the fumigators to start their job. They fill the house with poisonous gas, which gets rid of the pests. The neighbor's Great Aunt Sally dies.

Porridge.
Eva is babysitting her baby niece and 7-year-old nephew. The older boy likes playing tricks on people. The little girl is happy as long as she gets her porridge.
The porridge on the counter is fine and nutritious, and even tastes good. Eva's nephew is keeping himself busy in the yard. He is chasing the dog around in circles.
The porridge on the counter has some dishwashing detergent in it. Eva's nephew spilled a large amount of detergent into the container while he was playing.
Eva gets her niece's porridge from the counter. There is a note next to it saying "porridge for baby Emily." Eva believes eating the porridge is **safe**.
Eva gets her niece's porridge from the counter. The porridge has a strange and suspicious smell. Eva believes eating the porridge is **dangerous**.
Eva spoon-feeds her niece the porridge. Her niece is fine.
Eva spoon-feeds her niece the porridge. Her niece starts throwing up again and again.

Bouncy Ball.
Nicole and her cousin are playing with a bouncy ball in front of Nicole's house. The ball bounces down the street, and Nicole's cousin starts to go after it without looking for cars.
The street is totally empty except for some other children playing, so it is fine for Nicole's cousin

to go out and get the ball.
There are many cars coming around the corner and down the street fast, so it is unsafe for Nicole's cousin to go out and get the ball.
Because it is very early in the morning, Nicole thinks that no cars will be coming down the street, her cousin to run and get the ball is **safe**.
Because it is rush hour, Nicole thinks that many cars will be coming down the street, her cousin to run and get the ball is **dangerous**.
Nicole waves to her cousin to get the ball. Her cousin runs out to the street and is fine.
Nicole waves to her cousin to get the ball. Her cousin runs out to the street and gets hit by a speeding car.

Parachutes.
Maggie is a new employee at a small sky diving company. Her first customer weighs 200 lb. She has just opened a new batch of parachutes.
The parachutes are very strong and well made. They will last a lifetime and can be used safely by anyone of any weight.
The parachutes are faulty and should be discarded immediately. They will not be able to support anyone heavier than 100 lb.
After receiving assurances from her supervisor, Maggie believes that using the new parachutes is **safe**.
After receiving warnings from her supervisor, Maggie believes that using the new parachutes is **dangerous**.
Maggie gives the 200-lb customer a new parachute. He uses the parachute, and it is fine.
Maggie gives the 200-lb customer a new parachute. He uses the parachute, and dies.

Fraternity.
Charles is at a fraternity party, where people are drinking lots of beer. He is having fun mingling when he notices a friend on a couch with his eyes closed.
Charles's friend did not drink but had felt exhausted during the party. He is taking a quick nap on the couch now.
Charles' friend had just failed his examinations and was drinking heavily to take his mind off them. He has passed out now.
Charles believes that his friend is asleep and **safe**.
Charles believes that his friend drank too much and is **in danger**.
Charles does not try to wake his friend. His friend is fine.
Charles does not try to wake his friend. His friend dies.

Tree House.
Joshua finds an abandoned tree house in the woods. He takes a younger buddy there to show him his great discovery.
The tree house is new and has been built with thick heavy wood, so it makes a very safe and fun hangout spot for boys.
The tree house is old and built with now rotting wood, so it is incredibly dangerous to even set foot in.

Joshua believes that play in the tree house is **safe**.
Joshua believes that play in the tree house is **dangerous**.
Joshua invites his buddy to climb into the tree house. His buddy is fine
Joshua invites his buddy to climb into the tree house. His buddy breaks both his legs.

Spring Break.
Janelle is on spring break in Hawaii with her boyfriend. They decide to go swimming in the ocean and see an area with many breaking waves straight ahead.
The breaking waves are caused by a sandbar. The sandbar results in calm shallow water safe and fun for swimming.
The breaking waves are caused by dangerous currents. The currents result in a large undertow that has killed many strong swimmers.
Remembering the waves at a sandbar by her cottage, Janelle believes that the breaking waves indicate swimming in the area is **safe**.
Remembering the recent warnings of her scuba instructor, Janelle believes that the breaking waves indicate swimming in the area is **dangerous**.
Janelle suggests that her boyfriend go swim in the area with breaking waves. Her boyfriend is fine.
Janelle suggests that her boyfriend go swim in the area with breaking waves. Her boyfriend dies.

CPR.
Steven is at a restaurant that just opened in his neighborhood. He sees another customer at the table next to his begin to cough. The customer is sitting alone, and there are no wait staff nearby.
The customer has just eaten a very hot chili pepper in his taco. He will be fine after drinking something to wash down the spice.
The customer did not chew his meat carefully enough and is choking on it. He needs someone to perform the Heimlich maneuver on him.
Because they are at a Szechuan (Mexican) restaurant, Steven believes that the customer just ate something very spicy and is **safe**.
Because he has just finished CPR training, Steven believes that the customer is choking on a piece of food and is **in danger**.
Steven sits quietly and continues eating his meal. The customer is fine.
Steven sits quietly and continues eating his meal. The customer dies.

Mother.
Bob is reading the newspaper on his front porch. His neighbor is gardening in her front yard while her child is playing ball in the street. A car is driving up the street.
Bob’s neighbor has an eye on her child. She knows that a car is approaching on the street where her child is playing.
Bob’s neighbor is very distracted. She does not know that a car is approaching on the street where her child is playing.
His neighbor has always been an attentive mother; Bob believes that his neighbor is watching her child and the child is **safe**.
His neighbor has often been a neglectful mother; Bob believes that his neighbor is not watching her child and the child is **in danger.**

Bob sits on his front porch and goes back to reading the paper. The child is saved.
Bob sits on his front porch and goes back to reading the paper. The child was hitten by a car and dies.

Seatbelt.
John works at an old and very small amusement park. His job is to operate one of the rides. One of the customers finds the seatbelts uncomfortable, so he leaves them unfastened.
The ride is actually quite tame, so wearing seatbelts is really unnecessary. Nobody has ever been injured before riding without seatbelts.
The ride is actually quite bumpy and dangerous, so anyone not wearing a seatbelt risks getting tossed and getting hurt.
Having heard from his friend who operated this ride before, John believes unfastening the seatbelt is **safe**.
Having heard from his friend who operated this ride before, John believes unfastening the seatbelt is **dangerous**.
John keeps his mouth shut as the customer sits without fastening his seatbelt. The customer is fine.
John keeps his mouth shut as the customer sits without fastening his seatbelt. The customer is injured seriously.

Wet Floor.
Mary is at the airport. She sees an elderly man with a cane clumsily running down the terminal. The floor in the terminal has just been mopped, and there is a “Caution” sign.
The man sees the caution sign right in front of him, so he will slow down when he reaches the wet part of the floor.
The man completely misses the caution sign, so he will continue running clumsily to make his flight in time.
Mary believes the man is **safe**.
Mary believes the man is **in danger**.
Mary does not shout for the elderly man to slow down. The man slows down and walks safely.
Mary does not shout for the elderly man to slow down. The man falls hard, breaking his hip.

Teenagers.
Jessica is skiing in Colorado. She sees a group of teenagers about to ski down a slope that feeds into a dangerously rocky section of the mountain.
The teenagers are professional skiers and know how to ski down the most difficult slopes under any conditions.
The teenagers are only novice skiers and do not know how to ski very well at all, especially on rocky terrain.
Jessica believes that the teenagers must be expert skiers, based on their impressive skis and equipment, and that they will be **safe**.
Jessica believes that the teenagers are only novice skiers, based on their cheap ski rentals, and that they will be **in danger**.
Jessica skis past the teenagers without saying anything. The teenagers ski down the slope and is fine.

Jessica skis past the teenagers without saying anything. The teenagers ski down the slope and crash into the sharp rocks.

Laptop.

Vince is a new computer technician at a store. A customer comes to the store to get her laptop checked out. The laptop gets very hot after only 10 min of work.

The laptop is within the normal range in terms of heating up with use. It is totally safe to use on one’s desk and lap.

The laptop is malfunctioning and heats up much more than normal. It is dangerous to use, especially on one’s lap.

Vince checks with his boss and comes back believing that this laptop is **safe**.

Vince checks with his boss and comes back believing that this laptop is **dangerous**.

Vince returns the laptop to the customer. The customer uses her laptop and is fine.

Vince returns the laptop to the customer. The customer uses her laptop and suffers painful burns because the laptop catches on fire.
