# Supplementary figures and images for: Temporal Dynamics of the Integration of Intention and Outcome in Harmful and Helpful Moral Judgment
Source: Front Psychol. 2016 Jan 11;6:2022. doi: 10.3389/fpsyg.2015.02022 (PMC4708004; doi:10.3389/fpsyg.2015.02022)

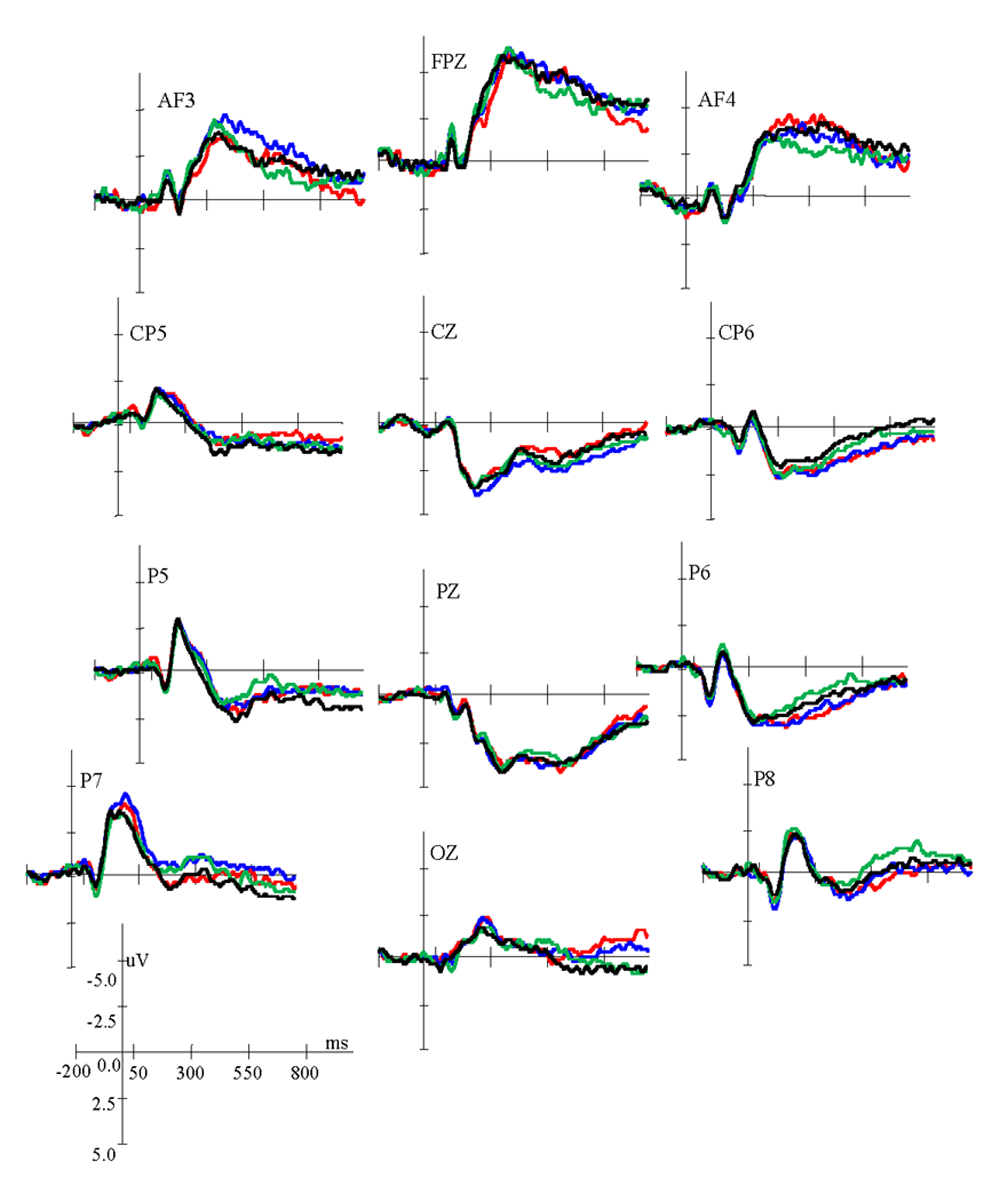

Supplement: Supplementary file 3 [file Image_1.TIF]

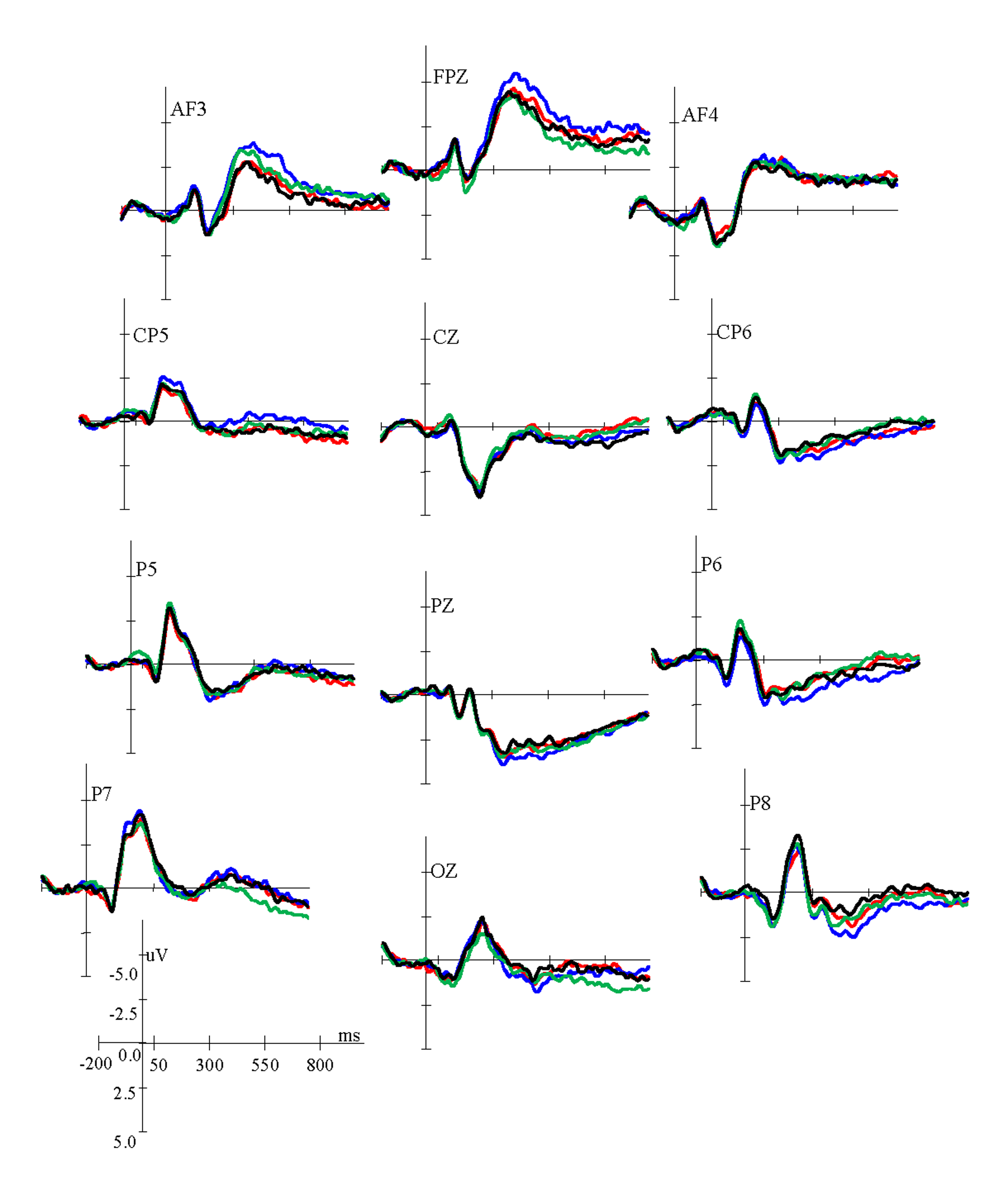

Supplement: Supplementary file 4 [file Image_2.TIF]

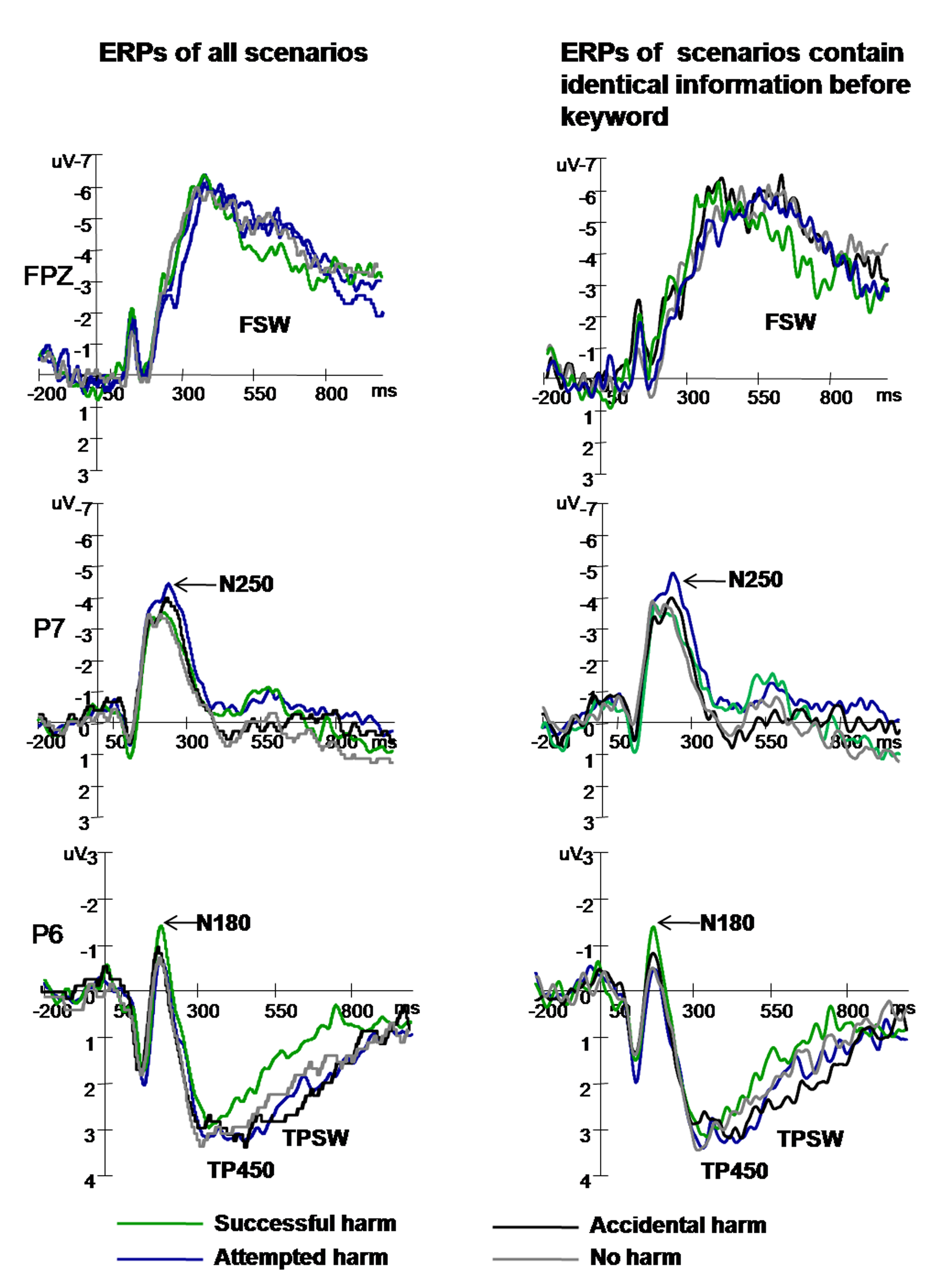

Supplement: Supplementary file 5 [file Image_3.TIF]
